# Sentiment analysis of tweets on alopecia areata, hidradenitis suppurativa, and psoriasis: Revealing the patient experience

**DOI:** 10.3389/fmed.2022.996378

**Published:** 2022-10-31

**Authors:** Irene Tai-Lin Lee, Sin-Ei Juang, Steven T. Chen, Christine Ko, Kevin Sheng-Kai Ma

**Affiliations:** ^1^Department of Radiology, Far Eastern Memorial Hospital, New Taipei City, Taiwan; ^2^Department of Anesthesiology, College of Medicine, Kaohsiung Chang Gung Memorial Hospital, Chang Gung University, Kaohsiung, Taiwan; ^3^Department of Dermatology, Harvard Medical School, Massachusetts General Hospital, Boston, MA, United States; ^4^Department of Dermatology, Yale University, New Haven, CT, United States; ^5^Department of Pathology, Yale University, New Haven, CT, United States; ^6^Center for Global Health, Perelman School of Medicine, University of Pennsylvania, Philadelphia, PA, United States; ^7^Department of Epidemiology, Harvard T.H. Chan School of Public Health, Boston, MA, United States; ^8^College of Electrical Engineering and Computer Science, Graduate Institute of Biomedical Electronics and Bioinformatics, National Taiwan University, Taipei, Taiwan

**Keywords:** sentiment analysis, Twitter, alopecia areata, hidradenitis suppurativa, psoriasis, mental health

## Abstract

**Background:**

Chronic dermatologic disorders can cause significant emotional distress. Sentiment analysis of disease-related tweets helps identify patients’ experiences of skin disease.

**Objective:**

To analyze the expressed sentiments in tweets related to alopecia areata (AA), hidradenitis suppurativa (HS), and psoriasis (PsO) in comparison to fibromyalgia (FM).

**Methods:**

This is a cross-sectional analysis of Twitter users’ expressed sentiment on AA, HS, PsO, and FM. Tweets related to the diseases of interest were identified with keywords and hashtags for one month (April, 2022) using the Twitter standard application programming interface (API). Text, account types, and numbers of retweets and likes were collected. The sentiment analysis was performed by the R “tidytext” package using the AFINN lexicon.

**Results:**

A total of 1,505 tweets were randomly extracted, of which 243 (16.15%) referred to AA, 186 (12.36%) to HS, 510 (33.89%) to PsO, and 566 (37.61%) to FM. The mean sentiment score was −0.239 ± 2.90. AA, HS, and PsO had similar sentiment scores (*p* = 0.482). Although all skin conditions were associated with a negative polarity, their average was significantly less negative than FM (*p* < 0.0001). Tweets from private accounts were more negative, especially for AA (*p* = 0.0082). Words reflecting patients’ psychological states varied in different diseases. “Anxiety” was observed in posts on AA and FM but not posts on HS and PsO, while “crying” was frequently used in posts on HS. There was no definite correlation between the sentiment score and the number of retweets or likes, although negative AA tweets from public accounts received more retweets (*p* = 0.03511) and likes (*p* = 0.0228).

**Conclusion:**

The use of Twitter sentiment analysis is a promising method to document patients’ experience of skin diseases, which may improve patient care through bridging misconceptions and knowledge gaps between patients and healthcare professionals.

## Introduction

Twitter, with over 320 million users, allows close to real-time exchange of ideas about current affairs through microblogging that consists of up to 280 characters (1, 2). The use of sentiment analysis on Twitter posts in medicine was first published in 2009 (3). This technique is a subfield of natural language processing whose aim is to automatically classify the expressed sentiment in texts (4). Since then, it has been widely applied to predict disease outbreaks (5–8), prescription of drugs and adverse drug reactions (9–13), patient satisfaction (14), public perceptions (15), and many others (16, 17). Other features of Twitter such as “likes” and “retweets” enable users to share, to show appreciation, and to propagate information that can be used to monitor trends in public perceptions. Sentiment analysis on this large dataset can provide an overview of the moods and emotional outcomes that are associated with specific diseases and physiological status. This method has the advantage of covering larger populations and geographic areas compared to traditional questionnaire-based methods (18).

As more and more people are turning to social media for health advice, understanding the sentiments of social media posts has become increasingly relevant (19, 20), as patients frequently report a lack of opportunity to express their psychosocial needs (21, 22). However, analysis of social media data in dermatology remains underutilized. Because dermatologic diseases are linked to numerous mental, physical, and emotional stressors that may not be easily captured during clinical visits, we believe that leveraging social media posts can help elucidate the subjective experience of dermatologic disorders. Thus, the objectives of this study were (1) to analyze the expressed sentiments in tweets related to alopecia areata (AA), hidradenitis suppurativa (HS), and psoriasis (PsO) (9–25); (2) to compare the sentiments related to skin disorders with that related to fibromyalgia (FM), a chronic musculoskeletal disease (26) without cutaneous manifestations; and (3) to validate the use of social media analysis for disease surveillance.

## Materials and methods

### Data collection

We used the standard Twitter application programming interface (API) to collect tweets containing keywords or tags for the diseases of interest. For HS, these included #Hidradenitis, #Suppurativa, #HidradenitisSuppurativa, #HSawareness, and “Hidradenitis Suppurativa”; for AA, “Alopecia areata”, #AlopeciaAreata, Areata, and AAAwareness; for PsO, Psoriasis and #Psoriasis; and for FM, Fibromyalgia, #Fibromyalgia, and #ChronicFatigueSyndrome. Searches using the Twitter API were case insensitive. There was a 180 requests per minute limitation with the standard API limits, which was considered sufficient for this study. Requests to the Twitter API were made through the “retweet” package in R Studio. Tweets that were publicly available and written in English were collected every day for 1 month (from April 1st, 2022, to April 30th, 2022). For each tweet, we obtained data on the date and time of creation, the user’s publicly displayed name, device type, tweet body text, and like and retweet status. A subgroup analysis of private/individual vs. public/organizational accounts (both types of accounts were open to public access) was carried out to determine whether discrepancies in illness experience exist.

### Sentiment analysis

To determine the expressed tones in each tweet, we used the AFINN lexicon developed by Finn Arup Nielsen and downloaded from the R “tidytext” package (27). The AFINN lexicon assigned a score between −5 (e.g., “bastard” and “twat”) and + 5 (e.g., “breathtaking” and “superb”) to each word, with negative scores suggestive of negative sentiment.

### Statistical analysis

The sentiment of each post was determined by the summation of the sentiment score of each word in the post. Independent *t*-tests were used to compare the means and standard deviations (SD) of sentiment scores between the diseases of interest. A *p*-value less than 0.05 suggested statistical significance. The data were collected and analyzed with RStudio 2022.07.1 + 554 for Mac (Boston, USA).

## Results

**FIGURE 1:**
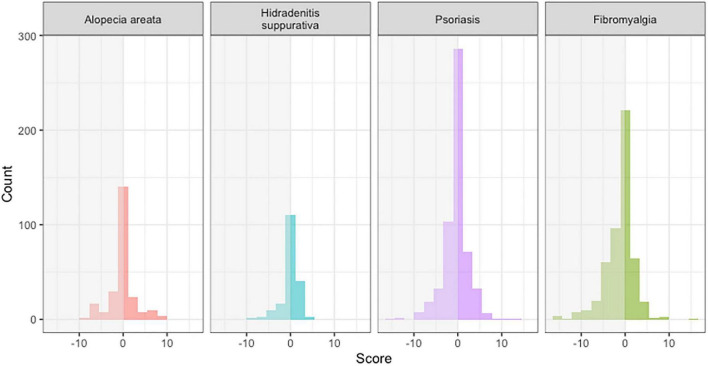
AFINN sentiment scores of tweets on different diseases.

We identified 243, 186, 510, and 566 tweets related to AA, HS, PsO, and FM, respectively. The mean sentiment score was −0.239 ± 2.90. The median and mode were 0. The average scores [mean ± SD (range)] for AA, HS, and PsO were −0.021 ± 3.29 (−10- + 10), −0.341 ± 2.41 (−10- + 6), and −0.308 ± 2.86 (−17- + 14), respectively (Figure 1). There was no significant difference among the three disorders (*p* = 0.482). There were 2–3 times more tweets from private accounts than from public accounts for all diseases. Posts from public accounts were significantly more positive (−0.128 ± 2.95 vs. −0.731 ± 3.21, *p* = 0.0008), especially for AA (0.729 ± 2.50 vs. −0.458 ± 3.61, *p* = 0.0082). On average, there were 0.656 ± 2.26 retweets and 5.77 ± 54.7 likes for each post.

### Words in negative and positive tweets on dermatologic disorders

**FIGURE 2:**
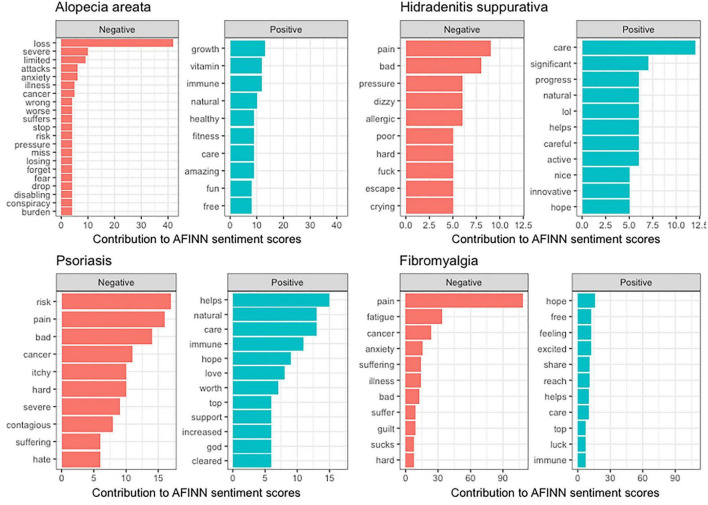
AFINN lexicon-graded positive and negative words commonly used in tweets about different diseases.

Figure 2 displayed the most frequent positive or negative words used in each specific disease. “Pain”, “bad”, and “hard” were used frequently in negative posts about HS and PsO; while “loss” was overwhelmingly presented in negative tweets on AA. “Anxiety”, “fear”, “wrong”, and “burden” were seen in posts on AA but not in posts on HS and PsO. Words expressing negative internal emotions such as “crying” were observed in posts on HS; words connoting external influence like “contagious” and “hate” were more commonly observed in posts on PsO than in posts on HS or AA. “Care” and “natural” were found in positive tweets related to all three diseases. Words describing a supportive system including “help”, “love”, “support”, and “god” were most frequently identified in positive posts on PsO.

### The sentiment of tweets on dermatologic disorders and fibromyalgia

510 tweets about FM were identified. Like skin disorders, “pain”, “bad”, and “hard” were commonly seen in negative posts on FM. Besides, emotional terms used in AA tweets, like “anxiety”, “suffering”, “guilt”, and “sucks”, contributed to a significant portion of negative posts on FM. The average sentiment score was significantly lower for FM (−1.11 ± 33.47) than for the three skin disorders, AA, HS, and PsO (−0.239 ± 2.90, *p* < 0.0001). Unlike skin disorders, tweets from public (−0.953 ± 3.70) or private (−1.170 ± 3.39) accounts expressed similar negative sentiment (*p* = 0.5969).

## Discussion

Findings of the present study provided an effective and efficient approach to measure sentiments surrounding AA, HS, and PsO via analysis on tweets. Words that have been given negative polarity, like “anxiety”, “pain”, and “crying”, are common in tweets related to AA, HS, and PsO. Sentiment regarding these three skin diseases is slightly polarized to the negative side, with less negative polarity compared to FM.

This study utilizes posts from popular social media to understand sentiments related to dermatologic disorders. The results seem to correlate well with previously documented psychiatric comorbidities. “Anxiety” was the most common emotional word in posts on AA. Patients with AA are particularly susceptible to generalized anxiety disorder (GAD) (28). A systematic review reported a 39–62% lifetime prevalence of GAD in patients with AA, giving an odds ratio (OR) of 7.28 compared to the general population. The ratio was higher than that for major depressive disorder (MDD) (OR = 5.87–6.77), social phobia (OR = 1.59–3.89), and paranoid disorder (OR = 4.4) (28). In contrast, for HS, words like “crying,” as well as aggressive words like “fuck” were commonly seen in tweets on HS, possibly reflecting the prevalence of bipolar disorders and MDD in this population. One meta-analysis on the psychiatric comorbidities of HS concluded that among the investigated psychiatric disorders, bipolar disorders (OR = 1.96) and MDD (OR = 1.75) were the most significantly increased comorbidities in patients with HS. Also in contrast to AA, for posts on PsO, we did not identify “anxiety” nor “bad” in the top 10 negative words. A recent meta-analysis reported a hazard ratio (HR) of 1.29–1.31 for anxiety in patients with PsO; on the contrary, the ratios were slightly lower than those found for AA and HS (29). The same study found that the OR for depression was 1.57 in patients with PsO (29). For comparison with all three skin disorders, tweets on FM were also examined. “Anxiety” and “guilt” were commonly used in negative posts on FM, for which patients with FM display a higher prevalence of GAD (20–80%) and MDD (13–63.8%) (30, 31). Thus, the approach adopted in the present study may be a powerful tool to conceptualize real-time emotional experience of dermatologic disorders, which may be used to predict or reflect their psychiatric comorbidities. Analyzing the psychological foundations of the affective lexicon allows for a better understanding of the emotional impact of diseases from patients’ perspectives and direct psychosocial interventions (32–34). Interestingly, the overall sentiment scores were neutral for the three dermatologic diseases and did not differ from one another. Despite their various health impact, previous studies suggested that the quality of life in dermatologic diseases was the most affected by the severity of diseases rather than the type of diseases (35–37). Our data may support this finding although we were not able to stratify sentiment scores by disease severity.

Besides emotional words, the dataset provided insight into other patient priorities. “Natural” and “care” were recurrent themes in all three diseases, suggesting growing interest in non-pharmacologic options. Words like “contagious” in tweets on PsO hinted at common misconceptions and could guide the development of future campaigns. Finally, a sentiment gap appeared between public and private accounts in tweets about skin disorders but not about FM. While a strong association between FM, depression, and anxiety is widely reported by lay media, many skin diseases were considered largely “cosmetic” and ignored for their emotional impact. Thus, this gap may reflect a failure of physicians and public organizations to identify occult emotional burdens. An empathetic and systematic approach may be beneficial and should be encouraged when caring for patients with dermatologic diseases. Furthermore, a previous study on tweets related to HS concluded that the analysis on social media data allowed the identification of some treatment beliefs not easily detected by traditional surveys (38). Collectively, these findings necessitated the presence of medical professionals and institutions to monitor and validate educational information on social media (39).

Despite continuous data collection for one month, the sample sizes were still small. In addition, we only analyzed one social media platform (i.e., Twitter), and therefore its external validity might be limited. A limitation specifically of Twitter API is the random selection of a number of tweets (set by users) during a period of time (set by users) from the pool of tweets using the specified hashtags/keywords. Twitter does not allow access to all qualified tweets with one search. Second, microbloggings on social media are usually used to express temporary emotions and may not adequately reflect long-term psychological status; and patients may be reluctant to publicly share either negative or positive experiences. Sentiment classification might fail when negation or irony are used. For example, profanity words can be used to modify a positive term, reversing their original polarity (14). Although irony may be indicated by emojis, previous studies did not show a significant improvement in sentiment classification with emoticons (40). Therefore, we did not include emojis in the analysis. Some people may use text embedded in images to trespass the 280-word limitation. These longer posts, which may be more personal, may be missed in the algorithm. Lastly, different sentiment lexicons can result in different results based on individual sensitivity and specificity. SentiStrength is another lexicon commonly used in health-related sentiment analysis (41, 42). That said, since AFINN was shown to have a similar or higher accuracy than SentiStrength, thus was preferred in this study (41).

## Conclusion

The use of sentiment analysis on tweets is a promising method that can reflect psychological comorbidities, illness experience, and public perceptions of patients with dermatologic disorders. This technique has the potential to improve patient care by bridging misconceptions and knowledge gaps between patients and medical professionals.

## Data availability statement

In the current study, the dataset regarding the reported results could be accessed via the publicly archived tool of Twitter application programming interface (https://help.twitter.com/en/rules-and-policies/twitter-api).

## Author contributions

ITL, SEJ, STC, CK, and KSM contributed to the conceptualization, data analysis, writing, and editing of the manuscript. All authors contributed to the article and approved the submitted version.

## Abbreviations

AA: alopecia areata
HS: hidradenitis suppurativa
PsO: psoriasis
FM: fibromyalgia
API: application programming interface.
